# Association between increased duodenal eosinophil count and functional dyspepsia

**DOI:** 10.1371/journal.pone.0351741

**Published:** 2026-06-16

**Authors:** Imteaz Mahbub, Bimal Chandra Shil, Sadeed Araf Reza

**Affiliations:** 1 National Gastroliver Institute and Hospital, Dhaka, Bangladesh; 2 Department of Gastroenterology, Sir Salimullah Medical College and Mitford Hospital, Dhaka, Bangladesh; Texas Tech University Health Sciences Center, UNITED STATES OF AMERICA

## Abstract

**Background:**

Functional dyspepsia (FD) is a common gastrointestinal disorder with multifactorial pathogenesis. Recent evidence suggests that duodenal eosinophilia may contribute to low-grade immune activation in FD. This study evaluated the association between increased duodenal eosinophil count and functional dyspepsia.

**Materials and methods:**

This case-control study was conducted in the Department of Gastroenterology, Sir Salimullah Medical College, Mitford Hospital, Dhaka, Bangladesh, from January to December 2022. Forty-six adult patients with functional dyspepsia diagnosed by Rome-III criteria were included as cases, while forty age- and sex-matched individuals without functional dyspepsia undergoing upper gastrointestinal endoscopy for other indications with normal endoscopic findings served as controls. Multiple biopsies were obtained from the second part of the duodenum. Formalin-fixed paraffin-embedded tissue sections were stained with hematoxylin and eosin. Eosinophils were counted manually by light microscopy in five randomly selected high- power fields (x 400 magnification), and the mean eosinophil count per high-power field (HPF) was calculated.

**Results:**

The mean duodenal eosinophil count was significantly higher in patients with functional dyspepsia compared with controls (23.98 ± 7.98 versus 15.63 ± 5.94 eosinophils/HPF, p <0.001). Duodenal eosinophilia (≥21 Eosinophils/HPF) was present in 69.6% of patients with functional dyspepsia compared with 17.5% of controls. Increased duodenal eosinophil count was significantly associated with functional dyspepsia (odds ratio 9.74, 95% confidence interval 3.50-27.08).

**Conclusions:**

Patients with functional dyspepsia demonstrated significantly greater duodenal eosinophil infiltration than controls, supporting the role of low-grade immune activation in its pathogenesis. Further multicenter studies with larger samples are required to clarify the clinical implications of duodenal eosinophilia in functional dyspepsia.

## Introduction

A range of symptoms in the flanks and epigastric area is referred to as dyspepsia [1]. Upper abdominal fullness, burning, flatulence, early satiation, nausea, vomiting, heartburn, regurgitation, frequent burping, and anorexia may be widespread as dyspeptic symptoms [2]. This set of symptoms may be divided into two subgroups: organic dyspepsia or functional dyspepsia [3]. Notable organic causes of dyspepsia include peptic ulcer disease, gastric or oesophageal cancer, GERD, Pancreatic or biliary disorder, intolerance to food, and some other systemic and infectious diseases [4]. On the other hand, functional dyspepsia may be defined as chronic or recurrent upper abdominal pain or discomfort in the absence of any known structural cause [5,6]. The symptoms of functional dyspepsia are thought to originate from the gastroduodenal region. The four symptoms are epigastric pain or burning, postprandial fullness, or early satiety [7]. According to the recently revised Rome criteria, functional dyspepsia is defined by the presence of persistent or recurrent dyspeptic symptoms for at least 3 months, with symptom onset at least 6 months before diagnosis, absence of structural disease explaining the symptoms on endoscopy, and lack of association of symptoms exclusively with defecation or stool irregularities.

The prevalence of functional dyspepsia ranges from 11% to 15% worldwide [5,6]. Prevalence varies across populations. The prevalence of functional dyspepsia in the US is 29.2% (with reflux) & 15% (without reflux), 23.8% in the UK, 14.7% in Norway, 17% in Japan, 24% in China, 30.4% in India, and 8.3% in Bangladesh [8–14]. Female gender and underlying psychological disturbances are important risk factors for functional dyspepsia [15]. The pathogenesis of functional dyspepsia is heterogeneous and multifactorial. Numerous systematic pathophysiological studies have identified factors that may contribute to the pathogenesis of functional dyspepsia. Major pathophysiologic mechanisms include dietary factors, motility disorders, sensorimotor dysfunction connected with hypersensitivity to mechanical and chemical stimuli, immune activation, elevated mucosal permeability in the proximal small intestine, disorders of the autonomic and enteric nervous system, infections, psychological factors, and genetics [16]. According to recent research, patients with functional dyspepsia exhibit low-grade immune activation, and increased numbers of immune cells have been found in the small intestine [17]. Duodenal eosinophilia has been investigated as one of the most important pathophysiologic mechanisms of functional dyspepsia in adults [18]. This may be explained by the higher concentration of eosinophils around submucosal plexus neurons in patients with functional dyspepsia, which is linked to neuronal dysfunction that drives symptom production [19].

However, data regarding the association between duodenal eosinophilia and functional dyspepsia remain limited in South Asian populations, particularly in Bangladesh. In addition, parasitic infestations, which are relatively common in developing countries, may influence duodenal eosinophil counts and potentially confound histopathological findings. Therefore, this study aimed to evaluate the association between increased duodenal eosinophil count and functional dyspepsia in a tertiary care hospital in Bangladesh while carefully excluding participants with evidence of parasitic infection.

## Materials and methods

### Study design and setting

A hospital-based case-control study was conducted at the Department of Gastroenterology, Sir Salimullah Medical College, Mitford Hospital, from January 2022 to December 2022.

### Study population

Adult patients diagnosed with functional dyspepsia according to Rome III criteria who attended the inpatient and outpatient departments were included as cases. Age and sex matched individuals without functional dyspepsia who underwent upper gastrointestinal endoscopy for indications such as evaluation of anemia, chronic diarrhea, or pre-endoscopic retrograde cholangiopancreaticography (ERCP) assessments and had normal endoscopic findings were included as controls.

A total of 46 cases of functional dyspepsia and 40 controls were included.

### Exclusion criteria

Patients with alarm features, including unintentional weight loss and progressive dysphagia, were excluded. Patients with a history of peptic ulcer disease, reflux esophagitis, gastrointestinal malignancy, pancreaticobiliary disease, recent gastrointestinal infection, inflammatory bowel disease, connective tissue disease, eosinophilic gastrointestinal disorder, drug reactions, parasitic infestations, or other conditions known to increase duodenal eosinophil count were excluded.

Patients with diabetes mellitus, renal dysfunction, chronic liver disease, thyroid disorders, severe psychiatric illness, or severe cardiac diseases were excluded. Individuals receiving non-steroidal anti-inflammatory drugs (NSAIDs), aspirin, corticosteroids, or medications known to affect eosinophil counts were also excluded.

### Clinical assessment and investigations

After obtaining informed written consent, demographic and clinical information were recorded in a structured data sheet. Functional dyspepsia was diagnosed using the validated Bengali version of the enhanced Rome-III questionnaire [20].

All participants underwent complete blood count, erythrocyte sedimentation rate, blood glucose, serum creatinine, liver function tests, thyroid-stimulating hormone, stool examination for ova and parasites, abdominal ultrasonography, and urea breath test for Helicobacter pylori.

Participants with anemia, peripheral eosinophilia (>500/mm³), positive stool examination for parasites, or any abnormal investigation suggestive of an organic disease were excluded.

### Endoscopy and biopsy procedure

Upper gastrointestinal endoscopy was performed using a standard forward endoscope (Olympus CV-170). Two to three biopsy specimens were obtained from the second part of the duodenum using standard biopsy forceps.

### Histopathological examination

Biopsy specimens were fixed in 10% buffered formalin, embedded in paraffin, sectioned at 5µm, and stained with hematoxylin and eosin. Histopathological examination was performed by an experienced gastrointestinal pathologist who was blinded to the clinical diagnosis.

Eosinophils were counted manually under light microscopy at x400 magnification in five randomly selected non-overlapping high-power fields (HPFs). The mean eosinophil count per HPF was calculated for each specimen. Duodenal eosinophilia was defined as ≥ 21 eosinophils/HPF [18].

### Statistical analysis

Continuous variables were expressed as mean ± standard deviation, while categorical variables were expressed as frequencies and percentages. Comparisons between groups were performed using the independent samples t-test and chi-square test or Fisher’s exact test where appropriate. Odds ratios with confidence intervals were calculated to assess the association between duodenal eosinophilia and functional dyspepsia. A p-value < 0.05 was considered statistically significant.

### Ethical consideration

Ethical approval was obtained from the institutional ethical review board of Sir Salimullah Medical College Mitford Hospital (Ref: SSMC/2021/247). Written informed consent was obtained from all the participants before inclusion.

## Results

### Baseline characteristics

Baseline demographic characteristics of the study participants are summarized in Table 1. A total of 86 patients were included in the study: 46 with functional dyspepsia and 40 controls. The mean age of participants in the functional dyspepsia group was 40.34 ± 16.22 years, compared with 40.15 ± 13.96 years in the control group. There was no significant difference in age distribution between the groups. (p = 0.997).

**Table 1:** Demographic characteristics of the study participants.

Parameter	Functional dyspepsia (n = 46)	Control (n = 40)	p-value
**Age (years)**			
18–30	15 (32.6%)	10 (25.0%)	
31–40	10 (21.7%)	13 (32.5%)	
41–50	8 (17.4%)	8 (20.0%)	0.997
51–60	6 (13.0%)	5 (12.5%)	
>60	7 (15.2%)	4 (10.0%)	
Mean ±SD	40.34 ± 16.22	40.15 ± 13.96	
**Gender**			
Male	21 (45.7)	19 (47.5)	0.863
Female	25 (54.3)	21 (52.5)	
**Monthly Income**			
<10,000	19 (41.3%)	9 (22.5%)	
10,000-30,000	22 (47.8%)	29 (72.5%)	0.066
>30,000	5 (10.9%)	2 (5.0%)	

Values are presented as numbers (%) unless otherwise indicated. p-values were calculated using the chi-square test

Females constituted 54.3% of patients with functional dyspepsia and 52.5% of controls. Gender distribution was comparable between the two groups (p = 0.863).

Most participants belonged to lower and middle-income groups. Although lower socioeconomic status was more common among patients with functional dyspepsia, the difference was not statistically significant (p = 0.066).

### Duodenal eosinophil count

**Fig 1:**
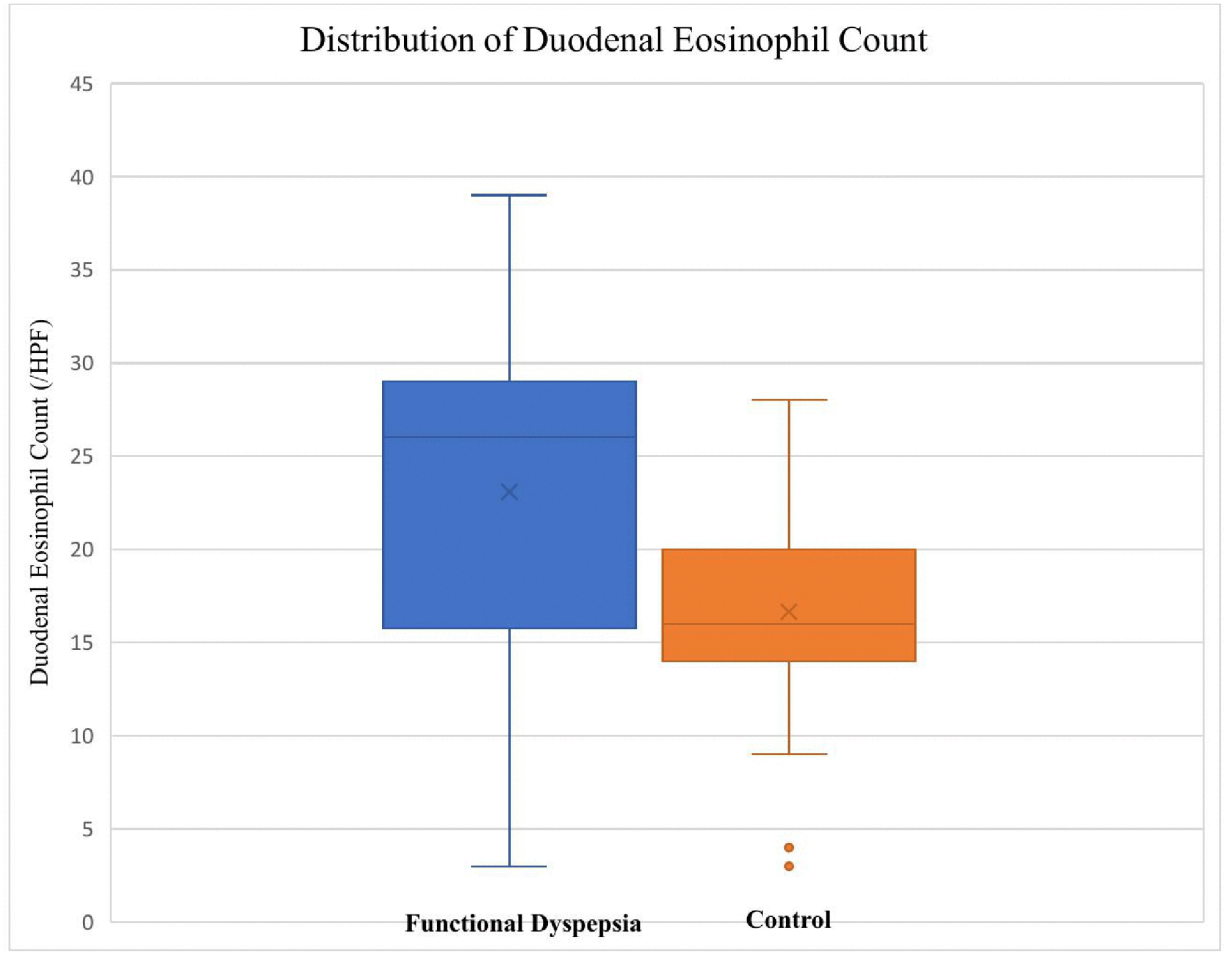
Box plot demonstrating the distribution of duodenal eosinophil counts in patients with functional dyspepsia and Controls.

The distribution of duodenal eosinophil counts among patients with functional dyspepsia and controls is illustrated in Fig 1, while comparative numerical data are summarized in Table 2. The mean eosinophil count in the second part of the duodenum was significantly higher among patients with functional dyspepsia than among controls (23.98 ± 7.98 vs 15.63 ± 5.94 eosinophils/HPF; p < 0.001).

**Table 2:** Comparison of mean duodenal eosinophil count between patients with functional dyspepsia and controls.

Site	Functional dyspepsia (n = 46) Mean ± SD (/HPF)	Control (n = 40) Mean ± SD (/HPF)	p-value
Second part of the Duodenum	23.98 ± 7.98	15.63 ± 5.94	<0.001

*p-values* were calculated using the independent samples t-test

#### Association between duodenal eosinophilia and functional dyspepsia.

The association between duodenal eosinophilia and functional dyspepsia is presented in Table 3. Duodenal eosinophilia (≥21 eosinophils/HPF) was identified in 32 (69.6%) patients with functional dyspepsia compared with 7 (17.5%) controls. The association was statistically significant (p < 0.001).

**Table 3:** Association between duodenal eosinophilia and functional dyspepsia (N = 86).

Duodenal eosinophil count (/HPF)	Functional dyspepsia (n = 46)	Control (n = 40)	p-value	OR	95% CI
≥ 21	32 (69.6%)	7 (17.5%)	<0.001	9.74	3.50-27.08
< 21	14 (30.4%)	33(82.5%)			

*Group comparison was performed using the chi-square test or Fisher’s exact test, where appropriate. Odds ratios (ORs) with 95% confidence intervals (CIs) were calculated to estimate the strength of association between duodenal eosinophilia and functional dyspepsia.*

Patients with increased duodenal eosinophil counts had significantly greater odds of having functional dyspepsia (Odds Ratio 9.74, 95% Confidence Interval 3.50–27.08).

## Discussion

Functional dyspepsia is a widespread morbidity throughout the world. The pathophysiology of functional dyspepsia has undergone significant changes over the past decade. It was previously thought to be only a brain-gut motility disorder without gut pathology. The new concept has been developed and is now considered a gut-driven disorder. Previous history of bacterial gastroenteritis, *H. pylori* infection, and psychosomatic factors are recognized as new factors behind the pathogenesis [17,21,22].

In our study, common age groups suffering from functional dyspepsia were 18–30 years and 31–40 years, with a mean age of 40.34 years. The mean age for the control group was 40.15 years. A small-scale study in Pune, India, found the mean age of the study population to be 38.25 years [23]. The common age group suffering from functional dyspepsia was 31–40 years in a study conducted in Balochistan, Pakistan [24]. To the contrary, two other studies conducted in China and Japan showed the 41–50 years and 50–59 years age groups as the peak age groups [11,12]. Our study had data similar to those of the Indian and Pakistani studies and dissimilar to those of the Chinese and Japanese studies. Possible explanations for the similarities and dissimilarities between these studies may include socio-cultural differences between South Asia and Eastern Asia. People from South Asia exhibit different socio-cultural behaviors. However, no significant difference in age was found between functional dyspepsia patients and the control group (p = 0.997)

A female preponderance (54.3%) was observed among patients with functional dyspepsia in this study. The female-male ratio was 1.2:1. A Korean study and a Japanese study conducted in 2012, based on Rome-III criteria, found a female-male ratio of 1.4:1 and 1.5:1, respectively, in patients with functional dyspepsia [25,26]. A different result was found in Pune. It found male predominance with a male-female ratio of 1.2:1 [23]. Female preponderance in the functional dyspepsia group may be due to differences in sex hormones, psychological distress, central signal via corticotropin-releasing factor (CRF), altered functional connectivity to amygdala, ghrelin, genetic factors, gut microbiome, and luminal factors [27].

A significant association between functional dyspepsia and lower socioeconomic status has been shown [28]. In our study, most patients with functional dyspepsia belonged to lower- and middle-income groups. An epidemiological study in China and another in Canada also found that lower socioeconomic groups were the most vulnerable [12,29]. The possible explanation of dyspeptic symptoms found in the low socioeconomic group may be due to their unhygienic food habits, ways of living, and stressful lives.

Thorough microscopic examination of all supplied biopsy specimen samples from both case and control groups was performed. Although some participants in the control group had clinical presentations such as chronic diarrhea and anemia, no significant histopathological abnormalities, such as villous atrophy, were identified.

In developing countries like Bangladesh, parasitic infestation remains an important confounding factor when evaluating tissue eosinophilia. Intestinal helminthic infection may cause peripheral and mucosal eosinophilia and, therefore, may falsely elevate duodenal eosinophil counts independent of functional dyspepsia. To minimize this bias, stool examination for ova and parasites was performed in all participants, and patients with parasitic infestation, recent Gastrointestinal infections, and peripheral eosinophilia were excluded from the study. Although these measures reduced the possibility of parasite-associated eosinophilia, subclinical or undetected infestations could not be completely excluded.

The mean eosinophil count in the second part of the duodenum was 23.98 ± 7.98 in patients with functional dyspepsia and 15.63 ± 5.94 in the control group. The difference between the two groups was statistically significant (P < 0.001). Looking at previous studies, in 2015, a Chinese study found the mean eosinophil count in the second part of the duodenum to be 24.8 ± 6.7 [30]. In our country, another notable study reported a mean eosinophil count of 22.5/HPF in patients with functional dyspepsia [31]. These two studies showed similarities with the current study’s findings. However, an Australian Cohort study reported a mean eosinophil count of 34.6 ± 16.9 in the second part of the duodenum of functional dyspepsia patients, which was higher than that in this study [18]. This difference is probably due to differences in lifestyle and dietary patterns between Western countries and South Asian countries.

Among patients with functional dyspepsia, 69.6% had duodenal eosinophilia, whereas 17.5% of the control group did. It was almost similar to the data of the study conducted in Pune, India. It found 65% of functional dyspepsia patients had duodenal eosinophilia [23]. The association between duodenal eosinophilia and functional dyspepsia was significant, with an odds ratio of 9.74 (95% CI 3.5049–27.0830, P < 0.0001). A significant association between early satiety and duodenal eosinophilia was found in the U.K. [32]. Moreover, the Bangladeshi study stated earlier also found a positive association between duodenal eosinophilia and functional dyspepsia patients [31].

Duodenal inflammation due to increased eosinophil count may contribute to functional dyspepsia. These findings raise the possibility that immune-targeted therapeutic strategies may warrant further investigation in selected patients with functional dyspepsia and duodenal eosinophilia.

A major strength of the study was careful exclusion of conditions known to influence duodenal eosinophil counts, including parasitic infestation, peripheral eosinophilia, allergic disorders, and organic gastrointestinal disorders. Furthermore, histopathological assessment was performed by a blinded gastrointestinal pathologist, minimizing observer bias.

This study had some limitations. The correlation between eosinophil count in tissue biopsy and the clinical picture and symptom severity was not evaluated. It was a single-center study with a small sample size. A large, multicenter study is required to assess the fact and increase the generalizability of the study nationwide. Although we tried to exclude all potential dietary factors that could affect the study, we might have missed some diets that could play minor roles in pathogenesis. Moreover, we could not exclude stress and anxiety among the participants.

### Conclusion

This study demonstrated a significant association between increased duodenal eosinophil count and functional dyspepsia in a Bangladeshi population. The findings support the hypothesis that low-grade duodenal immune activation may contribute to the pathogenesis of functional dyspepsia. Careful exclusion of parasitic infestation strengthened the reliability of the histopathological findings in the population. Further multicenter studies incorporating symptom severity assessment, dietary factors, and immunological markers are needed to determine whether duodenal eosinophilia may serve as a therapeutic target or biomarker in functional dyspepsia.

## Supporting information

S1 Dataset Dataset used for the analysis of duodenal eosinophil count and functional dyspepsia. (XLSX)

S2 Codebook Definitions and coding of variables used in the dataset. (DOCX)
