# Supplementary material for: Association between increased duodenal eosinophil count and functional dyspepsia
Source: PLoS One. 2026 Jun 16;21(6):e0351741. doi: 10.1371/journal.pone.0351741 (PMC13271515; doi:10.1371/journal.pone.0351741)
Supplement: S2 Codebook — (DOCX) [file pone.0351741.s002.docx]

| **Variable Name** | **Label** | **Type** | **Value Labels** |
| --- | --- | --- | --- |
| **SL_NO** | Serial Number | Numeric | *None* |
| **GROUPS** | Patient Group | Nominal | 1 = Functional Dyspepsia    2 = Non-Functional Dyspepsia |
| **AGE** | Age (Years) | Scale | *Continuous Value* |
| **AGEGR** | Age Group | Ordinal | 1 = ≤30    2 = 31–40    3 = 41–50    4 = 51–60    5 = >60 |
| **GENDER** | Gender | Nominal | 1 = Male    2 = Female |
| **SOCIO_EC** | Socio-economic Status | Ordinal | 1 = <10,000    2 = 10,000–30,000    3 = >30,000 |
| **EOSINOPH** | Eosinophil Count | Scale | *Duodenal Mucosa count* |
